# The gut microbiome stability is altered by probiotic ingestion and improved by the continuous supplementation of galactooligosaccharide

**DOI:** 10.1080/19490976.2020.1785252

**Published:** 2020-07-14

**Authors:** Chenchen Ma, Sanjeev Wasti, Shi Huang, Zeng Zhang, Rajeev Mishra, Shuaiming Jiang, Zhengkai You, Yixuan Wu, Haibo Chang, Yuanyuan Wang, Dongxue Huo, Congfa Li, Zhihong Sun, Zheng Sun, Jiachao Zhang

**Affiliations:** a College of Food Science and Engineering, Hainan University, Haikou, China; b Department of Human Nutrition, Food and Animal Science, University of Hawaii, Honolulu, HI, USA; c Single-Cell Center, CAS Key Laboratory of Biofuels and Shandong Key Laboratory of Energy Genetics, Qingdao Institute of BioEnergy and Bioprocess Technology, Chinese Academy of Sciences, Qingdao, China; d Key Laboratory of Dairy Biotechnology and Engineering, Ministry of Education P. R. C., Key Laboratory of Dairy Products Processing, Ministry of Agriculture and Rural Affairs China, Inner Mongolia Agricultural University, Hohhot, China

**Keywords:** probiotics, prebiotics, intestinal microbiome, *Lactobacillus plantarum* HNU082, galactooligosaccharide (GOS), single-nucleotide polymorphism (SNP), metagenome

## Abstract

The stable gut microbiome plays a key role in sustaining host health, while the instability of gut microbiome also has been found to be a risk factor of various metabolic diseases. At the ecological and evolutionary scales, the inevitable competition between the ingested probiotic and indigenous gut microbiome can lead to an increase in the instability. It remains largely unclear if and how exogenous prebiotic can improve the overall gut microbiome stability in probiotic consumption. In this study, we used *Lactobacillus plantarum* HNU082 (Lp082) as a model probiotic to examine the impact of the continuous or pulsed supplementation of galactooligosaccharide (GOS) on the gut microbiome stability in mice using shotgun metagenomic sequencing. Only continuous GOS supplement promoted the growth of probiotic and decreased its single-nucleotide polymorphisms (SNPs) mutation under competitive conditions. Besides, persistent GOS supplementation increased the overall stability, reshaped the probiotic competitive interactions with *Bacteroides* species in the indigenous microbiome, which was also evident by over-abundance of carbohydrate-active enzymes (CAZymes) accordingly. Also, we identified a total of 793 SNPs arisen in probiotic administration in the indigenous microbiome. Over 90% of them derived from *Bacteroides* species, which involved genes encoding transposase, CAZymes, and membrane proteins. However, neither GOS supplementation here de-escalated the overall adaptive mutations within the indigenous microbes during probiotic intake. Collectively, our study demonstrated the beneficial effect of continuous prebiotic supplementation on the ecological and genetic stability of gut microbiomes.

## Introduction

Probiotics are live microorganisms that can potentiate health benefits in the host when administered in appropriate quantities.^1^ The gut microbiome has long been considered a prospective target in the therapy for numerous diseases or general well-being.^2,3^ A variety of studies have demonstrated the beneficial effects of probiotics on the phenotypical modulation of the host gut microbiome.^4,6^ The commonly administered probiotics belong to the bacterial strains within genera *Lactobacillus* and *Bifidobacterium*. Probiotics usually exhibit shared advantageous properties,^7,8^ such as increased resistance against intestinal colonization by pathogenic microbes, restoration of the gut microbiome dysbiosis,^9^ and promotion of the growth of beneficial, indigenous microbes^10^ inhuman hosts.

Prebiotics are recognized for their capability to promote the fitness of probiotics or the indigenous intestinal microbiome, with both ecological and evolutionary ramifications.^11^ From the ecological perspective, an exogenous probiotic strain competes for nutrients and space with members already present in the indigenous microbiome. The appropriate supplementation of nutritional substrates as prebiotics for the gut microbial community could lower such competition and therefore enhance colonization of probiotic strains.^12^ The prebiotics such as fructooligosaccharide (FOS),^13^ xylooligosaccharide (XOS),^14^ galactooligosaccharide (GOS), and inulin^15^ have shown beneficial effects on probiotics *in vitro*. Our preliminary experiments found that galactooligosaccharide (GOS) used in the present study is the leading prebiotic for promoting the growth of Lp082. Previous studies have reported the molecular mechanism underlying GOS utilization by probiotics including *Lactobacillus* and *Bifidobacterium*; which highlighted the key roles of β-galactosidases (GH42 and GH2) in GOS metabolism.^16,18^ The highly abundant members of *Bacteroides* in the indigenous gut microbiome can also competitively utilize GOS, as they possess many carbohydrate-active enzymes that enable the digestion of a wide range of polysaccharides.^19^

Most of the reported benefits of prebiotics as well as the competition between exogenous and indigenous probiotics have been made from a microbial ecology and host physiology perspective.^20,22^ However, the competition between ingested probiotics and the resident microbiome within the gastrointestinal tract also results in a profound and previously unappreciated microbiome dynamic. First, unlike abiotic therapeutics, probiotics accumulate genomic mutations in a competitive environment.^23,24^ As a result, the indigenous intestinal microbiome could transiently fluctuate in abundance and genetically evolve rapidly to adapt to the invasion of probiotics.^25^ The direction of such co-evolution could eventually determine the ecological conditions in the gut microbiome and impact the therapeutic efficacy of probiotics treatments. It remains largely unclear if prebiotic supplementation can attenuate the probiotics-driven, genetic instabilities generated in the indigenous microbiome and how to most effectively administer the prebiotics from both the ecological and evolutionary standpoints.

**Figure 1.**
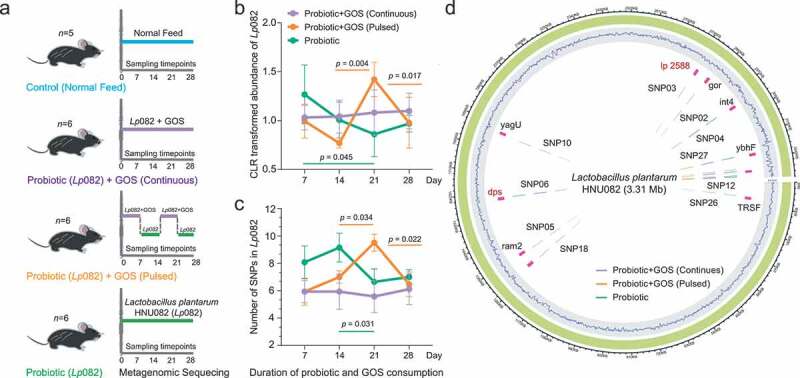
The experimental design and Lp082 adaptive evolution within host gut. (a) The experimental design. Control (*n* = 5 animals), PRO (*n* = 6 animals), GPC (*n* = 6 animals), and GPP (*n* = 6 animals). (b–c) The temporal dynamics of the relative abundance and the mutation frequency (SNPs) of Lp082 among the three groups, error bar: mean±SD. (d) Every SNP location was marked on the reference genome of Lp082.

In our previous work, we isolated a probiotic strain, *Lactobacillus plantarum* HNU082 (Lp082), from the traditional fermented seafood. We sequenced its whole genome^26^ and found encoded with an abundant number of carbohydrate-active enzymes and phosphotransferases. We also found that the bacteria exhibit a series of excellent probiotic characteristics.^27^ Therefore, it was developed as a model strain to systematically assess the ecological and evolutionary effects of probiotics on the gut microbiome, both in the presence and absence of prebiotic. In this study, we designed a 4-week microbiome study on mice using four treatment groups: Control, Probiotic, GOS+Probiotic Continuous (GPC), and GOS+Probiotic Pulsed (GPP) (Figure 1a). We longitudinally tracked the relative abundance of bacterial species-level compositions, single-nucleotide variants (SNV) profiles, and gene contents of these species in the fecal microbiome of mice. This study design allows us to specifically examine i) the impact of the supplementary GOS on the ecological and genetic stability of ingested probiotic Lp082 over time in the gut; ii) the impact of GOS supplementation on the overall stability of indigenous intestinal microbiome during probiotic consumption.

## Results

### The persistent GOS supplementation promote the colonization of the consumed probiotic and increased its genetic stability under the intestinal selective pressure

To profile the temporal dynamics of probiotics after consumption in each group, we first mapped the metagenomic sequences to the reference genome of Lp082 to calculate the relative abundance of this strain in each fecal microbiome. After probiotic intervention, the average relative abundance of the probiotic Lp082 was 0.025% among the 3 treatment groups (annotated from metagenomic reads), which only counted for less than millesimal indigenous microbes (Fig S1A). We found that, when GOS was not supplemented (i.e in PRO group), the relative abundance of the ingested probiotic decreased continuously over time possibly due to the selective pressure from indigenous gut microbiome (Figure 1b and Fig S1A). The probiotic abundance was more fluctuated over time, and highly correlated with the timing of the GOS supplement (Figure 1b) with the pulsed GOS supplement (i.e in GPP group). It dropped off from 7 to 14 d, went up from 14 to 21 d, then went down after 21 d. In contrast, its relative abundance was markedly stable over time (Figure 1b) under continuous supplement of GOS (i.e in GPC group). These results indicated that GOS supplementation can effectively enhance the colonization of the consumed probiotic in the intestinal microbiome.

We were next interested in delineating the impact of GOS supplementation on the population genetic stability of the probiotics under the selective pressure from the indigenous gut microbiome. Firstly, single-nucleotide polymorphism (SNP) on this probiotic genome in each fecal microbiome was identified by mapping the metagenomic sequences to the reference genome of Lp082 (Table S2). A total of 28 SNPs on this probiotic strain were annotated from all fecal microbiomes. When GOS was not supplemented, the total number of probiotic SNPs (averagely 8–9) was relatively higher during the first 14 d of colonization than that under GOS supplementation (Figure 1c–d) and was lowered after 14 d (averagely 6). In the case of pulsed GOS supplement, the number of adaptive mutations acquired by probiotics was associated with the timing of GOS supplementation. The persistent GOS supplement significantly decreased and stabilized the mutation frequency of consumed probiotic over the full course of ingestion. From the mutation frequency profiles, we found certain SNPs were significantly different in frequency between PRO and GPC groups (Fig S1C). These SNPs were mainly located on the gene *dps* encoding starvation-inducible DNA-binding protein, and the gene *lp_2588* encoding cell surface adherence protein.

### The continuous GOS supplementation stabilized the structural fluctuation of indigenous intestinal microbiomes induced by probiotic invasion

**Figure 2.**
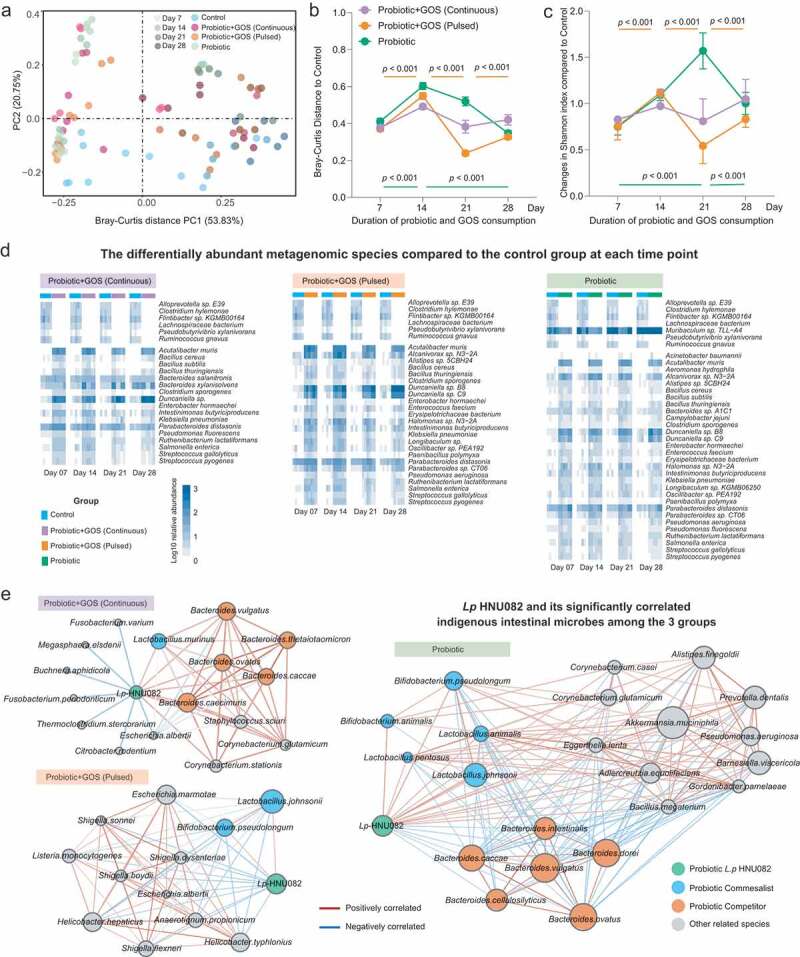
The indigenous intestinal microbiome response to the probiotic ingestion at the taxonomic level. (a) The PCoA plot based on the Bray–Curtis distance metric of species-level taxonomic profiles of fecal samples in each group. The points in different colors represented the samples in different groups, and the gradation of same color represented the samples in the same group but different time points. (b) The Bray–Curtis distance between samples in control and each of treatment groups at each time point, error bar: mean±SD. (c) The changes in taxonomic Shannon diversity compared to the control group at each time point, error bar: mean±SD. (d) The heatmaps showing significantly changed species-level taxa from control in each group. (e) The co-occurrence networks indicating the ecological relationships between Lp082 and indigenous intestinal species under each of treatments. The nodes in different colors represented by members in the community, i.e. Lp082, species positively correlated with probiotic, and species negatively correlated with probiotic. The edges are colored by sign and strength of correlation between a pair of nodes, which calculated based on the Spearman correlation coefficients.

We next sought to elucidate whether the response of indigenous intestinal microbiome to the ingested probiotics is GOS-supplementation-dependent. For that, the temporal changes in the beta diversity of indigenous gut microbiome based on the Bray–Curtis distance of the species-level taxonomic profiles (Figure 2a, Table S3) were quantified.

The probiotic ingestion spontaneously resulted in an increment in fecal alpha diversity (Figure 2c) and introduced the dramatic perturbations in the indigenous microbial community structure over time which is evident by Bray–Curtis distances to the control group (Figure 2b, c). In contrast, following the continuous supplementation of GOS, relatively high stability of gut microbiome over time was observed in the GPC group (Figure 2b, c). The pulsed GOS supplementation increased the probiotics-derived fluctuations. This result suggests that only continuous supplementation of prebiotics can reduce such microbiome instabilities due to probiotic invasion. We further identified the differentially abundant species to control at each time point in each group (Figure 2d). Among all probiotic-treated groups, we found the least number of species-level taxa in the GPC group tends to diverge in abundance from control over time. This further shows a relatively uniform microbiome configuration under continuous GOS supplementation.

Next, we constructed a co-occurrence network in each group to identify the ecological relationships between indigenous microbes and the consumed probiotic. Positive links represent commensalism or facilitation, while negative links represent antagonism or competition. From the network in PRO group (Figure 2e, right panel), the probiotic positively connected to *Bifidobacterium pseudolongum, Bifidobacterium animalis, Lactobacillus animalis, Lactobacillus pentosus*, and *Lactobacillus johnsonii*, while it showed a negative correlation to the species of the genus *Bacteroides*. Interestingly, the negative correlations between the Lp082 and *Bacteroides* species in the probiotic group turned to positive in GPC group (Figure 2e, left panel). The co-occurrence network was constructed based on the dynamics of the microbial abundance. The continuous GOS supplementation not only improved the colonization of the ingested probiotic in the indigenous gut microbial communities but also promoted the growth of indigenous *Bacteroides* species (such as *Bacteroides caccae, Bacteroides ovatus, Bacteroides vulgatus, Bacteroides thetaiotaomicron*, and *Bacteroides caecimuris*). Based on this observation, we concluded that the *Bacteroides* species were able to utilize the GOS, which lead to a strong competition between the Lp082 and *Bacteroides* species because of the similar carbohydrate utilization profile.

### The continuous GOS supplement featured changes in the carbohydrate-active enzymes (CAZymes) in the indigenous microbiome

**Figure 3.**
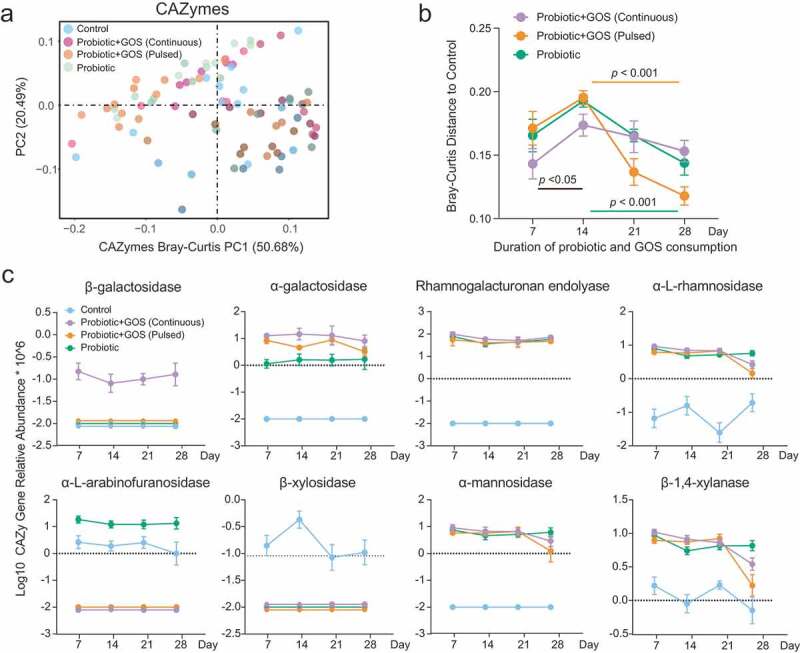
The consumption of the probiotic with prebiotic featured the temporal changes in the CAZymes of indigenous gut microbiome. (a) The PCoA plot based on Bray–Curtis distance of CAZymes profiles in the fecal microbiomes in each group. The points are colored by different treatment groups. (b) The Bray–Curtis distance between the control group and others based on CAZymes profiles at each time point, error bar: mean±SD. (c) The significantly changed CAZymes between the control and the other probiotic-treated (GPC, GPP, and PRO) groups, error bar: mean±SD.

To examine the impact of GOS supplement on the functional diversity in the indigenous fecal microbiome we characterized the temporal changes in the diversity of gut microbial CAZymes using the Bray–Curtis distance metric in all groups following probiotic treatments. The general disruption of the fecal CAZyme profiles by different probiotic administrations was evident by Bray–Curtis distances to the control group (Figure 3a, b). During the first 2 weeks, the Bray–Curtis distance of CAZymes in the fecal microbiomes increased consistently in each of the probiotics-treated groups which may be due to new exogenous bacteria introduced into the microbiome. Interestingly, a smaller Bray–Curtis distance of CAZyme profiles to the control group was observed under continuous GOS supplementation than that in other treatments. It shows that the CAZyme profiles of gut microbiome tend to be more similar to those in the control group during the first 2 weeks under continuous GOS supplement. After 2 weeks of probiotic colonization, the CAZyme profiles under all probiotic treatments were found to shift toward a control-group-like configuration.

The differentially abundant CAZymes between treatment groups at each time point were then identified (Figure 3c, Table S4). The relative abundance of the α-L-arabinofuranosidase and β-xylosidase decreased sharply in the GPC, GPP, and PRO groups as compared to control groups. The abundance of α-galactosidase, rhamnogalacturonan endolyase, α-L-rhamnosidase, α-mannosidase, and β-1,4-xylanase, however, increased significantly in the three probiotic-administrated groups. Notably, the β-galactosidase was maintained in a high abundance in the GPC group (Figure 3c). Therefore, we concluded that the consumption of probiotics and GOS leads to over-representation of certain key CAZymes in indigenous gut microbiomes that improved the overall functional capacity related to GOS utilization.

### The GOS supplement did not reduce the adaptive mutations within indigenous gut microbiomes due to probiotic consumption

**Figure 4.**
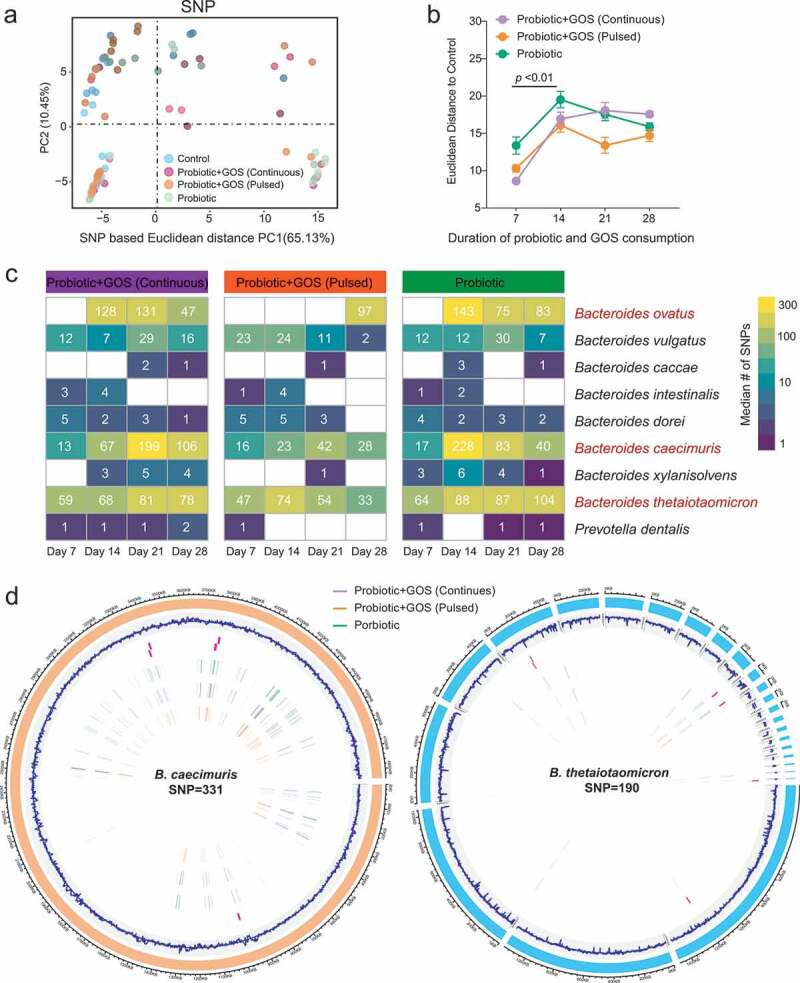
The GOS supplement did not reduce the adaptive mutations within indigenous gut microbiome due to probiotic consumption. (a) The PCoA plot based on the Euclidean distance of SNP profiles in each group. The points are colored by different treatments. (b) The Euclidean distance between the control group and others based on SNP profiles at each time point, error bar: mean±SD. (c) The heat map showing the median number of SNPs identified in each species in the probiotic-treated groups (GPC, GPP, and PRO) at each time point. (d) The distribution of identified SNPs in the genomes of the *Bacteroides caecimuris* and *Bacteroides thetaiotaomicron.*

Afterward, we examine the effects of GOS supplementation on the single-nucleotide variants (SNV) frequency of indigenous gut microbiomes during the ingestion of probiotics. For SNV profiling, 16 species that have sufficient coverage in at least 95% of the metagenomic samples were analyzed. Unexpectedly, 793 putative SNPs were identified in the probiotic-treated groups compared to control, suggesting the probiotic administration remarkably increased the overall mutations in the indigenous microbiome. From the PCA plot based on the SNV frequency table, prebiotic supplementation was observed to be a lesser association with SNV profiles (Figure 4a). The SNV differences related to time were more pronounced (Figure 4b). The SNV frequency of the indigenous microbes in all groups consistently increased within the first 2 weeks, and plateaued or slightly lowered after 2 weeks of probiotic consumption (Figure 4a–b). This result suggested that the GOS supplement did not reduce the overall changes in the genetic diversity within indigenous gut microbiomes in probiotic consumption.

Notably, among 793 SNPs identified in total, the vast majority (over 90%) was located in *Bacteroides* species (such as *Bacteroides ovatus, Bacteroides caecimuris*, and *Bacteroides thetaiotaomicron)* (Figure 4c, d, Table S5), which are also among the most abundant and prevalent species in the human gut. Genes under the adaptive evolution are mainly involved in carbohydrate utilization and cross-membrane polysaccharide importers such as transposase, integrase, carbohydrate-active enzymes, conjugative transposon protein, SusC/SusD outer membrane protein, and mobilization protein for plasmid transfer (Table S6). Interestingly, even though continuous GOS supplement changed the competitive relationship between indigenous *Bacteroides* and ingested probiotics (Figure 2e), both of them were still kept in a high frequency of adaptive mutations in the same gut ecosystem. It suggested that the evolution processes in the gut microbiome mediated by probiotic ingestion are faster, complex, and profound than is often assumed.

## Discussion

The stable gut microbiome plays a key role in sustaining host health, while the instability of gut microbiome also has been found to be a risk factor of various metabolic diseases. Healthy human gut microbiota is stable over long periods, where members of Bacteroidetes and Actinobacteria were able to be maintained over the course of more than 5 y.^28^ In contrast, that temporal shifts in the human gut microbiome are more frequent and extreme in Inflammatory Bowel Disease and acute myeloid leukemia patients as compared to healthy individuals. The high degree of temporal instability of stool and oral microbial diversity has been also observed in patients suffering from acute myeloid leukemia.^29,30^ The probiotics are commonly found to cause mild diarrhea in the early consumption stage. Such events can associate with the temporary instability of gut microbiome resulted from the intestinal colonization resistance to ingested probiotics.^31^ In this paper, we have highlighted the benefit of using GOS, and found that it not only promoted the growth of probiotics, but also buffered the possibly harmful response of the indigenous intestinal microbiome to probiotic invasion. Consequentially, this maintained the stability of the indigenous microbiome and reduced the side effects of probiotic intake.

The prebiotic has been found to promote the growth of probiotics, yet its impact on the stability of the gut microbiome remains largely unknown. Our study focused on the effects of prebiotic intake on the temporal stability of probiotics and the indigenous gut microbes in the probiotic consumption from both the evolutionary and ecological standpoints. The gastrointestinal tract is a battleground for residing microorganisms, where both indigenous and invasive microbes compete for the limited availability of nutrients such as carbohydrates, and space. From our microbial co-occurrence network analysis, we found that *Bacteroides* species have a strong negative link to the *Lactobacillus plantarum* when no prebiotics are supplied, suggesting a naturally occurring, strong, antagonistic relationship. Genome sequencing revealed that *Lactobacillus plantarum* and *Bacteroides* species (including *Bacteroides ovatus, Bacteroides vulgatus, Bacteroides caecimuris*, and *Bacteroides thetaiotaomicron*) consistently encode a large number of β-galactosidases. This suggests that they share similar carbohydrate utilization capabilities and an ecological niche with each other, which supports the strong negative link observation. Indeed, dietary GOS can enrich lactose-fermenting bacteria including *Lactobacillus* in the gut, along with the growth of certain *Bacteroides* spp.^32^ Coincidentally observed that their ecological relationship was eventually reshaped by the dual supplementation of probiotic and continuous GOS. Therefore, appropriate prebiotic supplementation with probiotics can attenuate the competitive interactions between the exogenous probiotic and the indigenous gut microbiome.

Influx of administered probiotics into the intestinal tract and subsequent competition with the indigenous microbiome leads to increasing instability of the gut microbiome.^33^ We observed that exogenous prebiotic carbohydrate reduced the deleterious changes induced by the probiotic invasion in the community composition of the indigenous gut microbiome. The intestinal microbial CAZymes also responded to the increased availability of nutrients. Intriguingly, we found that although GOS mitigates the intensive competition between them, the adaptive evolution within the genomes of both parties was still highly active throughout the whole study. More than 90% of these putative evolutionary changes were found in the indigenous microbial competitors of the probiotic Lp082, which likely promoted their competitive capacity and fitness in the gut. It strongly suggested that the common or even daily supplement of probiotics could rapidly become a strong driving force of both ecology and evolution in the indigenous gut microbiome within a relatively short timescale, that has been largely overlooked. Collectively, our study illuminated the effects of probiotic ingestion on the overall gut microbiome stability, as well as highlighted the GOS mediated co-evolution between the ingested probiotic and the indigenous gut microbiome. These findings provide novel sights about the beneficial effects of prebiotic supplementation with probiotics in the gut.

## Materials and methods

### Determining the optimal prebiotic for *Lactobacillus plantarum* HNU082

The optimal prebiotic of Lp082 was screened from GOS, XOS, FOS, stachyose, and inulin compared with the MRS medium based on the growth curve in the MRS medium *in vitro* and colony counting method. In brief, the screening medium was improved based on basal nutrient growth medium (peptone 10 g/L, beef extract 10 g/L, yeast extract 5 g/L, C_6_H_5_O_7_(NH_4_)_3_ 2 g/L, Tween 80 1 ml/L, CH_3_ COONa 5 g/L, K_2_HPO_4_ 2 g/L, MgSO_4_ 0.58 g/L, MnSO_4_ 0.25 g/L, pH adjusted to 6.2 with HCl solution) supplied with different prebiotics (20 g/L). The solid medium contained 2% agar. The growth curve showed that the growth capacity of Lp082 in the GOS-improved medium was like that of the MRS medium, while the number of viable counts was higher than that of the MRS medium. Thus, the GOS was selected as the optimal prebiotic of Lp082, Table S1.

### Animal experimentation and diets

Animal experimentation was carried at the Hainan University, Haikou, China following the guidelines approved by the Ethics Committee of the Hainan University. A total of 48 five-week-old male C57BL/6 mice were purchased from Hunan SJA Laboratory Animal Co., Ltd., which were housed at Hainan University, Haikou, China in speciﬁc pathogen-free conditions. After a 14-d adaptation period, all mice were randomly divided into 4 treatment groups (12 mice/group). These treatment groups were: Control, Probiotic (PRO), GOS+Probiotic Continuous (GPC), and GOS+Probiotic Pulsed (GPP). Mice were provided with the experimental diets for the next 4 weeks (Figure 1a). All the animals were provided with *ad libitum* feed and water. The Control group was fed with the commercially available feed (Hunan SJA Laboratory Animal Co.). The GPC group was fed with 8.6 Log10 CFU/day Lp082 and 20 mg/day GOS, while the GPP group was fed with 8.6 Log10 CFU/day Lp082 and 20 mg/day GOS (only on week 1 and week 3). The PRO group was fed with 8.6 Log10 CFU/day Lp082. The Lp082 and GOS were administered to mice by intragastric administration, along with normal saline, while the equal volume of normal saline was given to the control group.

### Fecal sample collection

Considering the possibility of accidental deaths during the trial, a total of 12 mice/treatment group were allocated at the beginning of the experiment. These 12 mice in each group were then further divided into 3 cages (4 mice/cage). The mice in each cage were numbered. Although 12 mice were fed in each group, we collected fecal samples from totally 5–6 mice (two out of four mice in each of three cages) under the same condition consistently in all time points of this experiment. To ensure that the fecal samples were fresh and collected at each time point and avoid any cross-contamination, we transferred each target mouse into a new clean cage for sample collection and return it to its original cage. Fecal samples were collected at d 7, 14, 21, and 28 from 5 mice (*n* = 5 for control) or 6 mice (*n* = 6 for PRO, GPC, and GPP) before gavage at 9 a.m. The fecal samples were snap-frozen in liquid nitrogen and stored at – 40°C until further analysis. After 4 weeks of experimental diet feeding, the mice were sacrificed by cervical dislocation. Every effort was made to minimize animal suffering.

### Fecal DNA extraction, shotgun metagenomic sequencing, and data quality control

The DNA was extracted from fecal samples by using the QIAamp® DNA Stool Mini Kit (Qiagen, Hilden, Germany) following the manufacturer’s instruction. The quality of the extracted DNA was determined by 0.8% agarose gel electrophoresis, and OD 260/280, while DNA concentration was measured by using NanoDrop 2000 (Novogene Company, Beijing, China). This was used for the shotgun metagenomic sequencing that was performed by Illumina HiSeq 2500 instrument in the Novogene Company (Beijing, China). The length of the DNA fragments after library preparation was approximately 300 bp. In the forward and reverse directions, 150 bp paired-end reads were generated. The reads were trimmed using Sickle software and were subsequently aligned to the mouse genome (GRCm38.p6) to remove the host DNA fragments.

### Identification of microbial species, functional genes, metabolic pathways, and the abundance of Lp082

The shotgun reads were assembled into contigs and scaffolds using MEGAHIT^34^ with the default parameter. For metagenomic species annotation, the Kraken 2.0^35^ and Bracken 2.5^36^ software were applied for taxonomic classification. HUMAnN2 was performed for metagenomic functional features and metabolic-pathway annotation based on the UniRef90 database.^37^ More information on the software and code should be found in “code availability.” Accordingly, we obtained the taxonomic, gene families, and metabolic-pathway profiles of the intestinal microbiome through the above analysis. Given the compositionality nature in the microbiome data,^38^ we further used CLR-transformed abundances based on the R package “composition”^39^ to perform univariate statistical analysis. Because the probiotic Lp082 had been whole-genome sequenced, we know every detail of its genome. When calculating the abundance of Lp082, firstly we constructed a reference genome database based on the whole genome sequence of the strain. The Lp082 reference genome was added into Kraken database, and the shotgun metagenomic sequencing reads of each sample were mapped to the Lp082 reference genome by running the Bracken 2.5 software, generating the relative abundance of Lp082 in each sample.

### Carbohydrate-active enzymes (CAZYmes) annotation and calculation of gene abundance

The dbCAN2^40^ software was used to identify and annotate CAZymes. In this regard, the assembled contigs by MEGAHIT were submitted and predicted to generate protein sequences using FragGeneScan v1.31.^41^ After protein prediction, Diamond v0.9.24^42^ was employed for fast blast hits in the CAZymes database (E value = 1e-102, based on CAZyDB on 08 August 2019). Then, the contigs were used to predict the functional genes with MetaGeneMark.^43^ Finally, a non-redundant gene catalog was constructed using CD-HIT.^44^ The abundances of genes were determined by aligning the reads back to the gene catalog using Bowtie2^45^ and SAMtools v_0.1.18.^46^ Subsequently, for any sample N, we calculated the abundance as follows:

Step 1: Calculation of the copy number of each gene:

(2) $$b_{i}=\frac{x_{i}}{L_{1}}$$

Step 2: Calculation of the relative abundance of gene i

(3) $$a_{i}=\frac{b_{i}}{\sum_{i}b_{i}}$$

*a_i_*: the relative abundance of gene i,

*b_i_*: the copy number of gene i from sample N,

*L_i_*: the length of gene i,

*x_i_*: the number of mapped reads.

The genome of Lp082 has been completely sequenced (Accession: PRJCA000348). The relative abundance of Lp082 was also calculated based on the above methods.

### Evolutionary analysis based on shotgun metagenomic data of gut microbiome

We next employed MIDAS (Metagenomic Intra-Species Diversity Analysis System) to profile the species-level SNP frequency and gene contents in the gut microbiome.^47^ Briefly, we constructed a reference genome database including the indigenous species in high abundance and the species closely related to Lp082. Then, the shotgun metagenomic sequencing reads were mapped to the database for the SNP calling on each of intestinal species using the same method as single genomic analysis. The SNPs identified from samples in the control group were set as the reference for the estimation of bacterial mutations of every single species at the following time points.

### Statistics statement

All statistical analyses were performed using R. Principle coordination analysis (PCoA) was performed in R using the ade4 package. The “zcomposition” package was used for CLR transformation. The differentially abundance analysis on each microbial feature in the taxonomic and functional profiles was tested with Wilcoxon rank-sum test or Kruskal–Wallis test on CLR-transformed data. A microbiome feature is considered differentially abundant at *p* < .05 (the data were corrected for multiple testing, and the *p* value was the adjusted *p* value, which is also considered as q value). The package “ggplot2” was used for generating bubble and box-and-whisker plots. The line chart was constructed using Graphpad Prism 6.07. The heatmap was constructed using the “pheatmap” package, and the evolutionary dynamics were built using the “ggmuller” package. The microbial ecological networks were inferred by Spearman’s rank correlation coefficient from the metagenomic sequencing data and visualized in Cytoscape (Version 3.7.1).

## Supplementary Material

Supplemental Material

Supplemental Material

## Data Availability

The sequence data reported in this paper have been deposited in the NCBI database (metagenomic sequencing data: PRJNA608079). More details about the figure construction and code can be found in Github: https://github.com/HNUmcc/HNU082_GOS_project
