# Supplementary material for: The gut microbiome stability is altered by probiotic ingestion and improved by the continuous supplementation of galactooligosaccharide
Source: Gut Microbes. 2020 Jul 14;12(1):1785252. doi: 10.1080/19490976.2020.1785252 (PMC7524268; doi:10.1080/19490976.2020.1785252)
Supplement: Supplemental Material [file KGMI_A_1785252_SM1004.pdf]

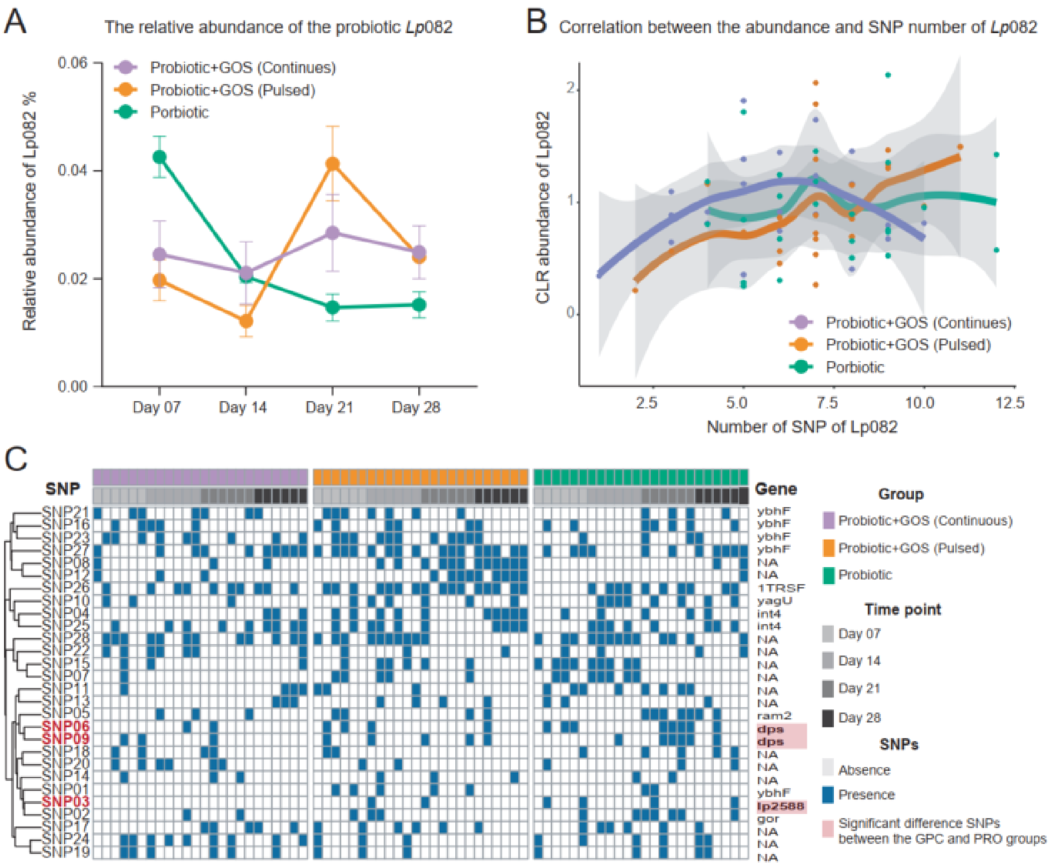

Figure S1. A) The relative abundance of probiotic Lp082. B) The correlation between the abundance and SNP number of Lp082. C) The heatmap showing the presence or absence of SNPs detected in all samples. The samples in each group are shown in columns, while the detected SNPs with gene annotation are displayed in the rows.

Table S1. The impacts of different prebiotics on the growth of Lp082

The impacts of different prebiotics on the growth of *Lp*082								
	**0h**	**2h**	**4h**	**6h**	**8h**	**10h**	**12h**	**14h**
**Galactooligosaccharide (GOS)**	0.05	0.15	0.43	1.37	1.76	1.88	1.95	1.97
	0.06	0.16	0.42	1.37	1.76	1.88	1.97	1.97
	0.06	0.15	0.43	1.36	1.76	1.89	1.97	1.96
**GOS Average OD600**	0.06	0.16	0.43	1.37	1.76	1.88	1.97	1.97
**Xylooligosaccharide (XOS)**	0.06	0.18	0.57	1.08	1.22	1.59	1.68	1.67
	0.07	0.19	0.56	1.09	1.23	1.58	1.66	1.68
	0.07	0.18	0.58	1.07	1.24	1.60	1.64	1.67
**XOS Average OD600**	0.07	0.18	0.57	1.08	1.23	1.59	1.66	1.67
**Fructo-oligosaccharide (FOS)**	0.08	0.09	0.27	0.59	1.16	1.49	1.64	1.71
	0.07	0.10	0.28	0.60	1.15	1.48	1.64	1.70
	0.07	0.09	0.27	0.58	1.14	1.48	1.64	1.69
**FOS Average OD600**	0.07	0.09	0.27	0.59	1.15	1.48	1.64	1.70
**Stachyose**	0.08	0.19	0.52	0.75	0.89	0.93	0.99	1.04
	0.07	0.18	0.54	0.75	0.90	0.94	1.00	1.04
	0.08	0.19	0.53	0.76	0.89	0.94	0.99	1.04
**Stachyose Average OD600**	0.07	0.19	0.53	0.75	0.89	0.94	0.99	1.04
**Inulin**	0.07	0.18	0.56	0.82	1.11	1.15	1.16	1.16
	0.07	0.19	0.56	0.83	1.11	1.16	1.16	1.16
	0.06	0.19	0.55	0.82	1.11	1.16	1.16	1.16
**Inulin Average OD600**	0.07	0.19	0.56	0.82	1.11	1.16	1.16	1.16
